# Frequency-dependent signaling in cardiac myocytes

**DOI:** 10.3389/fphys.2022.926422

**Published:** 2022-09-02

**Authors:** Payam Haftbaradaran Esfahani, Jan Westergren, Lennart Lindfors, Ralph Knöll

**Affiliations:** ^1^ Department of Medicine Huddinge, Karolinska Institutet, Huddinge, Sweden; ^2^ Wendelsbergs beräkningskemi AB, Mölnlycke, Sweden; ^3^ Advanced Drug Delivery, Pharmaceutical Sciences, BioPharmaceuticals R&D, AstraZeneca, Mölndal, Sweden; ^4^ Department of Medicine, Integrated Cardio Metabolic Centre (ICMC), Heart and Vascular Theme, Karolinska Institute, Stockholm, Sweden; ^5^ Bioscience Cardiovascular, Research and Early Development, Cardiovascular, Renal and Metabolism (CVRM), BioPharmaceuticals R&D, AstraZeneca, Mölndal, Sweden

**Keywords:** cell geometry, contraction frequency, cardiac myocyte, cardiomyocyte, sarcomere, signaling, mathematical modeling, simulation

## Abstract

**Background:** Recent experimental data support the view that signaling activity at the membrane depends on its geometric parameters such as surface area and curvature. However, a mathematical, biophysical concept linking shape to receptor signaling is missing. The membranes of cardiomyocytes are constantly reshaped due to cycles of contraction and relaxation. According to constant-volume behavior of cardiomyocyte contraction, the length shortening is compensated by Z-disc myofilament lattice expansion and dynamic deformation of membrane between two adjacent Z-discs. Both morphological changes are strongly dependent on the frequency of contraction. Here, we developed the hypothesis that dynamic geometry of cardiomyocytes could be important for their plasticity and signaling. This effect may depend on the frequency of the beating heart and may represent a novel concept to explain how changes in frequency affect cardiac signaling.

**Methods:** This hypothesis is almost impossible to answer with experiments, as the *in-vitro* cardiomyocytes are almost two-dimensional and flattened rather than being in their real *in-vivo* shape. Therefore, we designed a COMSOL multiphysics program to mathematically model the dynamic geometry of a human cardiomyocyte and explore whether the beating frequency can modulate membrane signal transduction. Src kinase is an important component of cardiac mechanotransduction. We first presented that Src mainly localizes at costameres. Then, the frequency-dependent signaling effect was studied mathematically by numerical simulation of Src-mediated PDGFR signaling pathway. The reaction-convection-diffusion partial differential equation was formulated to simulate PDGFR pathway in a contracting sarcomeric disc for a range of frequencies from 1 to 4 Hz. Results: Simulations exhibits higher concentration of phospho-Src when a cardiomyocyte beats with higher rates. The calculated phospho-Src concentration at 4, 2, and 1 Hz beat rates, comparing to 0 Hz, was 21.5%, 9.4%, and 4.7% higher, respectively.

**Conclusion:** Here we provide mathematical evidence for a novel concept in biology. Cell shape directly translates into signaling, an effect of importance particularly for the myocardium, where cells continuously reshape their membranes. The concept of locality of surface-to-volume ratios is demonstrated to lead to changes in membrane-mediated signaling and may help to explain the remarkable plasticity of the myocardium in response to biomechanical stress.

## Introduction

Cells are constantly exposed to mechanical stress by forces that are generated by gravity, organ motion, blood flow, extracellular cell-to-cell and cell-to-matrix interactions, and intracellular traction. These mechanical forces have been shown to influence a broad spectrum of cellular behaviors, from proliferation and differentiation to transcriptional responses and gene expression.

Mechanotransduction is a fundamental biological process, by which cells sense mechanical stimuli, integrate them, and convert them into biochemical signals, inducing downstream cellular responses (Martino et al., 2018). Because biomechanical force can be transmitted through the cytoskeleton, microtubules, and actin stress fibers, its propagation time is much shorter than the diffusion of molecules in signaling pathways. This means that the cells respond rapidly to their dynamic environment. Defective mechanotransduction has been associated with numerous diseases from all fields of medicine, including cardiology, dermatology, gastroenterology, nephrology, neurology, oncology, ophthalmology, orthopedics, pediatrics, pulmonary medicine, reproductive medicine, and urology. Abnormal mechanotransduction can be caused by alterations in, or malfunctions of, one or more of the entities involved in the force sensing and conversion process, namely, the extracellular matrix (ECM), cell surface receptors, the cell cytoskeleton, and the various molecules associated with the signaling cascade that occurs in the cytoplasm or nucleus.

Signaling activity at the membranes depends on global cell geometry parameters, such as the cellular aspect ratio (Haftbaradaran Esfahani et al., 2019), size (Nowak et al., 2021), membrane surface area, and membrane curvature (Haftbaradaran Esfahani and Knoll, 2020b). A recent paper by Rangamani et al. (2013) has presented a “curvature-dependent mechanism of transient receptor activity enhancement,” but its relevance for biology and medicine remains unclear. The authors showed how the enhancement of kinase activity in downstream signaling pathways could be mediated by transient enhancement of receptor activity, following increased ligand binding in the curved plasma membrane areas of elliptic cells with increasing eccentricity. They demonstrated that information contained in the cell’s shape could be transformed into measurably different MAPK phosphorylation levels in the nucleus. Matter exchange by lateral diffusion, between areas of high and low curvature at the plasma membrane, will eventually equilibrate the ligand-induced inhomogeneity of receptor activity. However, when fission of membrane areas with high curvature occurs, this matter exchange cannot take place. This could lead to stabilization of the enhanced interaction with cytoplasmic effectors at the membrane interface of the endocytosed vesicle.

It has been well documented that the effects of global cell geometry influence signaling activity and differentiation (Haftbaradaran Esfahani and Knoll, 2020b), but the local effects are still poorly understood. Cells use various local membrane curves to perform cellular functions, such as filopodia to form adhesions, endosomes for intracellular signaling, and caveolae to moderate membrane tension. Invagination or protrusion of local membranes has been shown to respond differently to stimulus, by increasing cell eccentricity. This is due to the transient assembly of the activated receptors in microdomains with higher curvature. This transient inhomogeneity in the distribution of activated receptors arises from a local imbalance between reactions and the diffusion of soluble ligands and receptors in the plane of the membrane (Rangamani et al., 2013).

When protrusion occurs, there is higher accessibility to external ligands, since the reactive volume to surface ratio is higher for ligand-receptor binding. On the other hand, when invagination occurs, the volume to surface ratio for the ligand-receptor binding is limited, although the release of the intracellular receptor-bound signaling molecule is easier.

It has recently been shown by Lou et al. (2018) that curvature sensing proteins were engaged upon manipulating the membrane by nanostructures. In addition to membrane curvatures, they showed that gene expression was dysregulated after nano-structural deformation of nuclear envelope. In another study, Elliott et al. (2015) investigated the closed loop relation between regulation of myosin II and fluctuation of local membrane curvature in endothelial cells.

In heart, cardiac myocytes (CMs) beats with cycles of contraction and relaxation. In each beating cycle, sarcomeres, the contractile unit of CM between two adjacent Z-discs, shorten and elongate. Since the cytosol is incompressible, the cytosol is isovolumetric and the cell volume is constant during a cycle of contraction-relaxation. Therefore, when the sarcomere shortens, the Z-disc lattice spacing expands. Correspondingly, when the sarcomere relaxes (i.e., elongates), the Z-disc lattice spacing compresses [for a review please see (Knoll et al., 2011)]. In the transverse sections, Z-discs show two distinct structural states: the “small square” form and the “basket weave” form. In the classical studies of fixed muscle tissues, it has been proposed that the small square and the basket-weave forms represent the relaxed and the active contracted states, respectively (Williams et al., 2013; Malingen et al., 2020), but this view has recently been challenged (Oda and Yanagisawa, 2020; Burbaum et al., 2021). Interestingly, it has been shown that in the contracted state, the lattice was not expanded that much, leading to a decrease in Z-line lattice volume (Irving et al., 2000; Toh et al., 2006). Thus, the membrane needs to be stretched (Kamkin et al., 2000; Samarel, 2005) to sustain the normally constant cytosolic volume during contraction by pushing the cytosol towards the cell membrane (Malingen and Daniel, 2020; Cass et al., 2021; Powers et al., 2021).

Since the cell volume is constant, the periodic relative motion of neighboring Z-discs leads to the advection of cytosol molecules. Moreover, the curvature of CM membrane fluctuates periodically with a constraint to isovolumetric cytosol (Knoll, 2015). Accordingly, we developed the hypothesis that dynamic geometry of CMs could be important for CM plasticity and signaling. This effect may depend on the frequency of the beating heart and may represent a novel concept to explain how changes in frequency affect cardiac signaling. This hypothesis is almost impossible to answer with experiments, as the *in-vitro* cultured CMs are nearly two-dimensional and flattened rather than being in their real *in-vivo* shape. Moreover, it is very demanding to prove the hypothesis by culturing adult human CMs for several days, transduce and pace them (Wright et al., 2020). Therefore, comprehensive mathematical models are critical for predicting geometry-dependent cellular signaling. These models could be implemented on platforms that have been specifically designed for modeling cell biological systems, such as VCell (virtual cell) software or on general multi-physics platforms like COMSOL Multiphysics.

In our case, we designed a COMSOL program (COMSOL, 2021) to mathematically model and numerically simulate the dynamic geometry of CM and explore whether the frequency of contraction can modulate membrane signal transduction.

Src kinase is an important component of cardiac mechanotransduction (Schlaepfer and Hunter, 1996; de Cavanagh et al., 2009). In this paper, we first presented that Src kinase mainly localizes at costameres, a crucial site for CM survival and growth (Samarel, 2005). In addition, costameres have substantial roles in mechanically-induced signaling in CMs (Martino et al., 2018). It has been previously demonstrated that Platelet-Derived Growth Factor (PDGF) induces Src activation (Thomas and Brugge, 1997; Rangamani et al., 2013; Lu et al., 2017; Fan et al., 2022). We visualized the spatio-temporal activation characteristics of Src kinase upon PDGF stimulation, by introducing a fluorescent Src activation biosensor into the cultured neonatal rat CMs (NRCMs). The Src biosensor, named KRas-Src, works based on the FRET technique to report Src activation (Liu et al., 2014). We introduced the KRas-Src FRET reporter into the NRCMs through adenoviral delivery. We then stimulate the NRCMs by PDGF and acquire the FRET images using a confocal microscope at 5 s and 5, 10, and 20 min after PDGF treatment. The FRET image analyses were performed afterwards to measure the spatio-temporal Src activity in NRCMs.

**FIGURE 1:**
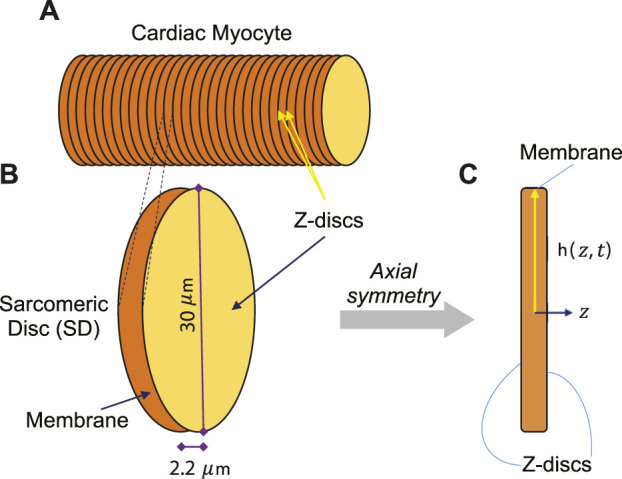
**(A)** The adult human CM was assumed to have cylindrical shape, consisting of several symmetric Sarcomeric Discs (SDs). **(B)** One contracting SD of an adult human CM was assumed to be disk-shaped with 30 µm diameter and 2.2 µm thickness in relaxed state. **(C)** One SD was presumed to have axial symmetry to simplify the simulations to two-dimensional longitudinal section.

We call each bunch of laterally-registered sarcomeres as a “Sarcomeric Disc” (SD) (see Figures 1A,B) and assume signaling is the same for all SDs. Therefore, we mathematically modeled a SD and investigated our central hypothesis by simulating the effect of frequency of contraction on the activation of Src kinase through PDGF signaling in the COMSOL-modeled SD of an adult human CM. In order to monitor the concentration of activated Src at different locations in the SD at different times, transport of all the included substances in combination with reactions were simulated numerically.

To create a dynamic multiphysics model of membrane signaling, we assumed that a CM is like a cylinder, consisting of several symmetric SDs. Since SDs are similar repeating units, we can simply model this membrane signaling at the membrane of one SD instead of modeling a whole CM. During a contraction-relaxation cycle of an adult human CM, the sarcomere length (SL) is shortened from 2.2 μm at the relaxed state to 1.6 μm at the contracted state, widening the Z-disc radially. Although, the SL-lattice spacing relationship has been investigated in *in-vivo* (Toh et al., 2006) and *in-vitro* (Irving et al., 2000) animal models, the amount of lattice expansion in an *in-vivo* adult human CM has not been studied yet. However, if one assumes a similar relationship, this would indicate that Z-disc lattice of a SD would be expanded about 2 μm at the contracted state in an *in-vivo* adult human CM.

## Materials and methods

### Src activation biosensor

We used the FRET technique to monitor the activity of Src kinase experimentally. The FRET-based Src activation biosensor (Wang et al., 2005) consists of ECFP (Enhanced Cyan Fluorescent Protein) and YPet, an improved version of YFP (Yellow Fluorescent Protein), linked together by the SH2 domain (from c-Src), a flexible linker and the Src-specific substrate peptide, derived from c-Src substrate p130Cas. The KRas-Src biosensor was constructed by fusing 14 KRas-prenylation sequences (KKKKKKSKTKCVIM) to the C-termini of Src biosensor (Pan et al., 2019). The donor:acceptor (ECFP:YPet) stoichiometry is fixed to 1:1. The activity of Src kinase causes a conformational change which modifies the distance between donor and acceptor, resulting a FRET change. When Src is at the resting state, the conformation of the Src construct is such that ECFP and YPet are juxtaposed, leading to a high FRET. When Src is active, it phosphorylates the substrate domain at tyrosine sites; therefore, the Src reporter is unfolded, inducing the separation of ECFP and YPet, reducing the FRET emission. To increase the delivery efficiency of KRas-Src biosensor, we used an E1-defective adenoviral vector of KRas-Src FRET reporter.

### Cell isolation, cell culture and adenoviral transduction

NRCMs were isolated and purified as previously described (Haftbaradaran Esfahani and Knoll, 2020a). Hearts were rapidly excised from two-day-old Sprague-Dawley rats and tissue samples were collected from the dorsal of the apex of the left ventricle. NRCMs were isolated from the tissue using the Neonatal Heart Dissociation Kit (Miltenyi Biotec, Bergisch Gladbach, Germany). Isolated cells were resuspended in DMEM:M199 (4:1), supplemented with 10% horse serum, 5% fetal bovine serum and 1% penicillin/streptomycin (10,000 U/ml) (Thermo Fisher Scientific, Waltham, MA, United States). The suspension was pre-plated for 2 h in 10 cm^2^ uncoated cell culture flasks (Nunc, Thermo Fisher Scientific) to remove the fibroblast and endothelial cells, by allowing non-CMs to attach to the surface of the culture flask. Enriched NRCMs were seeded at a density of 7 × 10^3^ cells/mm^2^ on 35 mm MatTek glass bottom dishes (Part No. P35G-1.5), pre-coated with 2 µg/cm^2^ fibronectin. 24 h after plating, the plating medium were replaced with serum-free maintenance medium. Then, the cultured NRCMs were incubated at 37°C with 10 MOI KRas‐Src adenovirus for 48 h.

### FRET imaging of Src activity

After 72 h in culture, we carried out the FRET imaging of Src activity upon PDGF stimulation of serum-starved NRCMs. To avoid autofluorescence of the culture medium containing phenol red, maintenance medium was washed and replaced with 1 ml HBSS (Hank’s Balanced Salt Solution), supplemented with 20 mM HEPES and 2 g.l-1 D-glucose. Then, we placed the culture dish on the stage of a Leica SP5 inverted confocal microscope. We saved the coordinate of a well-transduced, spontaneously contracting NRCM. However, the frequency of contraction should be less than 0.25 Hz to assure the bias from contraction is negligible. Then, we immediately treated NRCMs with 30 ng/ml PDGF. In a temperature-controlled imaging chamber, the selected NRCMs were imaged with a 63X/1.4 oil-immersion objective. Fluorophores were excited with a 457 nm laser. The ECFP and FRET channels were recorded simultaneously at 5 s and 5, 10, and 20 min after PDGF treatment and the fluorescence intensity ratios for ECFP/FRET were plotted as a function of time.

### Image processing and FRET analysis

To quantify the spatio-temporal FRET changes of the Src reporter, we used two-channel ratiometric approach (i.e., ECFP/FRET ratio) for time series analysis. Therefore, we acquired a pair of ECFP and FRET images for each time course (i.e., 5 s, 5, 10 and 20 min) of the experiment. The intensity values of the acquired images range from 0 to 255. The image processing procedure for FRET analysis was performed using Fiji ImageJ (Schindelin et al., 2012). We first deducted the image noise average from all pixels of the image. Then, we subtracted the background, surrounding the cell of interest from both ECFP and FRET images. We analyzed Src kinase activity upon PDGF stimulation solely at costameres, since we found that Src kinase mainly concentrated at the costameres in CMs (i.e., pixels belong to costameres have higher fluorescence intensity). Additionally, by just considering the costameres, the bias from cytosolic autofluorescence is reduced drastically. Then, we generated a binary mask by using an intensity threshold of 90 on FRET images, since we found that most of the costamere pixels could be picked out by this threshold. As some pixels which do not belong to costameres could pass the threshold, we used a median filter, called “Despeckle,” on the binary thresholding mask to remove these undesired pixels. Despeckle replaces each pixel with the median value of its 3 × 3 surrounding pixels. We then performed the “AND” operation of the tailored binary mask and the original image to generate the masked image. We calculated the FRET ratio of each time course by dividing the masked ECFP image by masked FRET image, pixel by pixel. The obtained FRET ratios of time series were finally normalized by the value of the first time course (i.e., at t = 5 s).

### Mathematical model for membrane and intracellular gradients in a beating sarcomeric disc

The adult human CM was assumed to have cylindrical shape, consisting of several symmetric SDs (Figure 1A). Since sarcomeres are similar repeating units, we modeled one contracting SD (Figure 1B). We presumed axial symmetry of one SD to simplify the simulations to two-dimensional longitudinal section (Figure 1C).

The shape of the SD changes with time when the Z-discs are moved towards each other to contract the sarcomere and consequently the entire cell. As the SD shortens and in order to maintain the constant intracellular volume, the Z-discs expand to a bigger diameter and the cell membrane stretches. We let the model to compute the amount of membrane stretch at each time point, constrained to isovolumetric cytosol. The SD radius is a function of the z coordinate and time and is denoted h(z,t) (Figure 1C). The time dependence is modelled as a cosine function with *T* being the period of the contraction.

$$h\left(z,t\right)=15\;\text{µm}+\left(2\;\text{µm}+0.985\;\text{µm}\cdot \left(1-{\left|\frac{z}{z_{0}\left(t\right)}\right|}^{3}\right)\right)\cdot \frac{1-cos\frac{2\pi t}{T}}{2}$$ (1)

Note that the location of the Z-discs varies with time. The maximum radius is found at z=0 and the minimum radius at the Z-discs where $z=\pm z_{0}\left(t\right)$ .

$$z_{0}\left(t\right)=1.1\;\text{µm}-0.3\;\text{µm}\cdot \frac{1-cos\frac{2\pi t}{T}}{2}$$ (2)

**FIGURE 2:**
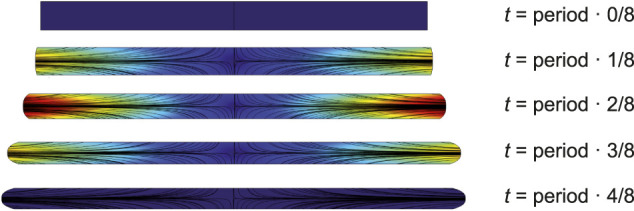
Dynamic evolution of SD geometry at 0, $\frac{1}{8}$ , $\frac{1}{4}$ , $\frac{3}{8}$ , $\frac{1}{2}$ of each contraction period. The black contours represent velocity fields of cytosol. The color code shows the magnitude of velocity from dark blue, equals to 0, to dark red as maximum value.

The shape change of the SD is shown in (Figure 2). As a simplification, there are no organelles or other obstacles inside the sarcomere, so the cytosol can flow freely. The shape change causes cytosol flow back and forth towards the cell membrane. Given the membrane shape changes in Eq. 2, the velocity field $\vec{u}$ of the cytosol was calculated using Navier-Stokes equations. No-slip boundary conditions are applied. Thus the velocity of the cytosol at the domain wall are zero relative to the surface. The stretching of the Z-discs was uniform in radial direction.

The finite-element mesh was generated to divide the model into small elements, over which the Navier-Stokes equations were solved. The velocity field of cytosol at different times is shown in (Figure 2).

The motion of the different substances in the cytosol is governed by diffusion and advection according to the diffusion-advection equation

$$\frac{\partial c}{\partial t}=D\Delta c-\nabla \cdot \left(\vec{u}c\right)$$ (3)

where c is the concentration of a specific substance, D is its diffusivity and $\vec{u}$ is the time-dependent velocity field of cytosol. The Δ is the Laplace operator and ∇ is the Del operator. The membrane receptors are free to move at the membrane by two-dimensional diffusion,

$$\frac{\partial c_{2D}}{\partial t}=D_{2D}\Delta c_{2D}$$ (4)

where $c_{2\text{D}}$ is the concentration with respect to the area and $D_{2\text{D}}$ is a two-dimensional diffusivity. The boundary constraint at the membrane—Z-disc interface is ∂c/∂z=0 for all substances. In addition, it is assumed that there is no resultant cytosolic flow through the Z-disc during the contraction-relaxation period.

Reactions that take place in the cytosol only add an extra reaction term to Eq. 3.

$$\frac{\partial c}{\partial t}=D\Delta c-\nabla \cdot \left(\vec{u}c\right)+R$$ (5)

and analogously for reactions that take place in the membrane only, an extra term is added to Eq. 4.

$$\frac{\partial c_{2D}}{\partial t}=D_{2D}\Delta c_{2D}+R_{2D}$$ (6)

Reactions involving substances both in the membrane and in the cytosol are written as a flux from the membrane (production) or towards the membrane (consumption). Thus, these reactions are described by boundary conditions where the normal flux towards the membrane is

$$F=\vec{n}\cdot \left(D\nabla c\right)$$ (7)

where $\vec{n}$ is the normal vector from the membrane into the SD. This flux equals the reaction rate, i.e., $F=R_{2D}$ . For the rest of the boundaries, i.e., the Z-discs, the transport of the all substances are zero. The differential equations are solved with the Finite Element Method (FEM) using the software COMSOL. The time-dependent solver used is the Backward Differentiation Formula (BDF).

**FIGURE 3:**
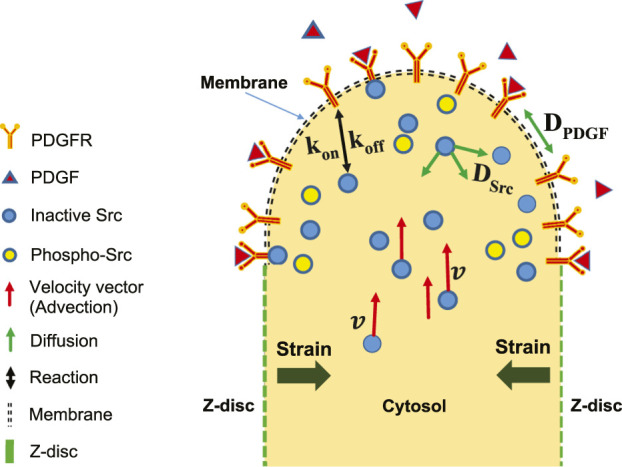
Schematic of reaction-diffusion-advection model in the upper half of two-dimensional longitudinal section of one SD. Shortening of SD causes strain. Extracellular PDGF can bind to membrane receptors, PDGFR. The intracellular inactive form of Src can bind to PDGFR. The inactive and activated forms of Src are free to diffuse in any direction, whereas PDGFR is limited to diffuse along the membrane plane, with the diffusion coefficients of $D_{Src}$ and $D_{PDGFR}$ respectively. The concentration of inactive and activated Src vary due to diffusion and reaction, but also vary due to advection (shown by velocity vector field v). Advection is a consequence of strain, which is due to SD contraction.

A schematic of reaction-diffusion-advection system for the concentration of Src, is shown in (Figure 3). The lists of reactions and kinetic parameters of the simulated PDGF signaling are reported in Table 1 and Appendix 1. Moreover, the initial concentrations and diffusion coefficients of the molecules interacting in simulated PDGF signaling are provided in Tables 2, 3. In addition, it is presented in Table 3 whether 2D or 3D equations were applied to each molecules. In our numerical model, extracellular PDGF binds PDGF receptor (PDGFR), forming PDGF-PDGFR complex which reacts with inactive form of Src in cytosol and activates Src.

**TABLE 1:** Reactions and kinetic parameters for modeling Src activation upon PDGF stimulation (Rangamani et al., 2013).

Name	Reaction	Reaction type	Kinetic parameters
PDGF binds PDGF-Receptor	PDGF + PDGFR ↔ PDGF-PDGFR	Binding	$K_{on}=1\;\mu M^{-1}s^{-1}$
			$K_{off}=0.01\;s^{-1}$
Src phosphorylation	Src + PDGF-PDGFR ↔ Phospho-Src	Enzymatic	$K_{M}=1\;\mu M$
			$K_{cat}=0.1\;s^{-1}$
Src inactivation	Phospho-Src + Csk-active ↔ Src	Inactivation	$K_{M}=1.5\;\mu M$
			$K_{cat}=0.3\;s^{-1}$
Csk activation	Csk → Csk-active	Activation	$K_{f}=0.001\;s^{-1}$

**TABLE 2:** Initial concentrations for modeling Src activation upon PDGF stimulation (Rangamani et al., 2013).

Name	Initial concentration	Compartment
PDGF	30 ng/ml	Extracellular space
PDGF Receptor	250 molecules.µm-2	Membrane
Src	1 µM	Cytoplasm
Csk	0.1 µM	Cytoplasm

**TABLE 3:** Diffusion coefficient, compartment and dimension of equation that applied to the molecules, interacting in the PDGF signaling (Rangamani et al., 2013).

Name	Diffusion coefficient $\left(\mu m^{2}/s\right)$	Compartment	Dimension of equation
Csk	10	Cytoplasm	3D
Csk-active	10	Cytoplasm	3D
Src	10	Cytoplasm	3D
Phospho-Src	1	Cytoplasm	3D
PDGF	1	Extracellular space	N/A
PDGFR	0.041	Membrane	2D
PDGF-PDGFR	0.041	Membrane	2D

## Results

### Experimental exploration of spatio-temporal activation of Src in neonatal rat CMs

We first studied the spatio-temporal activation of Src kinase upon PDGF stimulation in NRCMs. Src kinase is normally present in its inactive conformation. Upon PDGF treatment of CMs, the extracellular PDGF binds to the membrane-bound receptor, PDGFR, and activates it. The activated transmembrane PDGFR phosphorylates (i.e., activates) the intracellular inactive-Src by recruiting PTP (Protein Tyrosine Phosphatase). The activated Src (phospho-Src) can again be inactivated by a non-receptor tyrosine kinase Csk (C-terminal Src Kinase) (Matsuoka et al., 2004; Okada, 2012).

As described in the Methods section, we studied the characteristics of Src activation in NRCMs using ECFP/FRET ratiometric analysis. Since ECFP is very prone to bleaching, it has to be exposed by the least possible laser exposure in order that we can neglect photobleaching in FRET analysis. Therefore, the intensities of ECFP and FRET channels were recorded at four time courses that are 0+, 5, 10, and 20 min after PDGF treatment.

**FIGURE 4:**
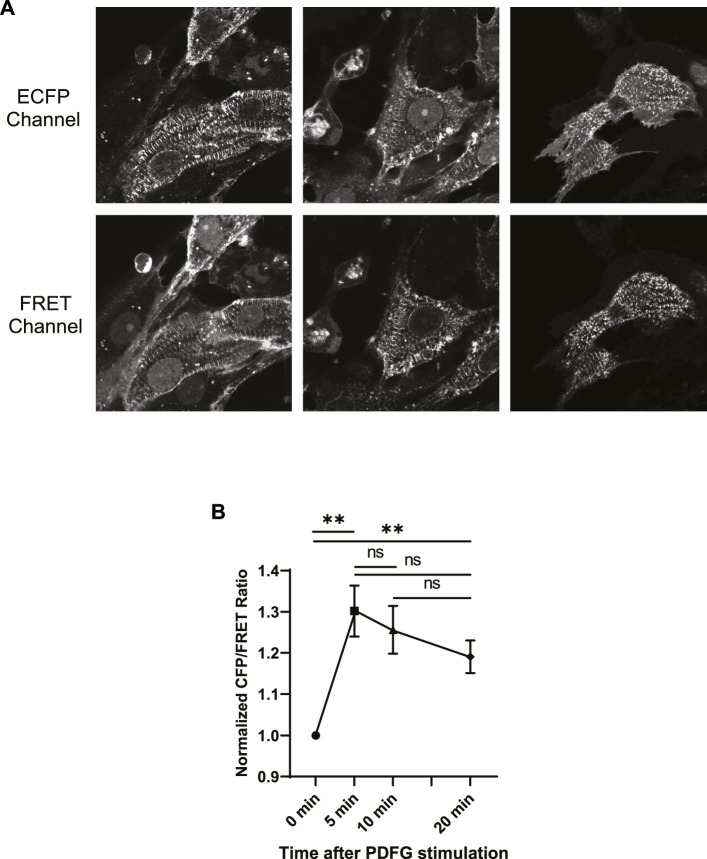
**(A)** Fluorescent signal images of ECFP and FRET emission channels acquired from three ECFP-YPet transduced NRCMs at t = 0+ of PDGF stimulation. **(B)** Normalized ECFP/FRET intensity ratios at t = 0+, 5, 10 and 20 min after PDGF treatment. Although, the ECFP/FRET values of time points t = 5, 10, and 20 min were not significantly different (unpaired two-tailed t-test), compared to each other, the ECFP/FRET values of these time points were significantly higher, compared to t = 0+. The ECFP/FRET measurement was performed at the costameres pixels, using the approach described in the materials and methods section.

ECFP and FRET images of some NRCMs were shown in (Figure 4A). The figure illustrates the highest fluorescence intensities emitted from costamere sites, suggesting that Src kinase is mainly localized at the costameres in CMs. Although, lower fluorescence was detected from other regions of the cell, we analyzed Src kinase activity solely at costameres, according to importance of the costameres in CM signaling. In addition, the autofluorescence bias is predominant when analyzing lower fluorescence signal, detected from non-costamere regions.

We then calculated the fluorescence intensity ratios of ECFP/FRET for each time course and plotted the normalized ratio as a function of time in (Figure 4B). Although, the ECFP/FRET values of time points t = 5, 10 and 20 min were not significantly different (unpaired two-tailed t-test), compared to each other, the ECFP/FRET values of these time points were significantly higher, compared to t = 0+. This indicates Src activation upon PDGF stimulation, consistent with previous reports in different cell types (Thomas and Brugge, 1997; Rangamani et al., 2013; Fan et al., 2022). The ECFP/FRET measurement was performed at the costameres pixels, using the approach described in the materials and methods section. Accordingly, we probed costamere to mathematically examine our hypothesis.

### Mathematical examination of the hypothesis

#### Frequency-dependent Src activation in adult human cardiac myocytes

Different frequencies of heart beating and various patterns of contraction and relaxation are common in almost all heart diseases. Variations in pattern of CM beating lead to dynamic geometry of SDs. Accordingly, we hypothesized that changes in the contraction frequency of CM lead to dynamic geometry of SDs. The dynamic geometry of SD generates concentration gradient for membrane and intracellular molecules, which modulate membrane signal transduction. As it is almost impossible to examine our hypothesis using *in-vivo* and *in-vitro* systems, we mathematically simulate Src-mediated PDGF signaling by a finite-element COMSOL model, as described in methods section.

**FIGURE 5:**
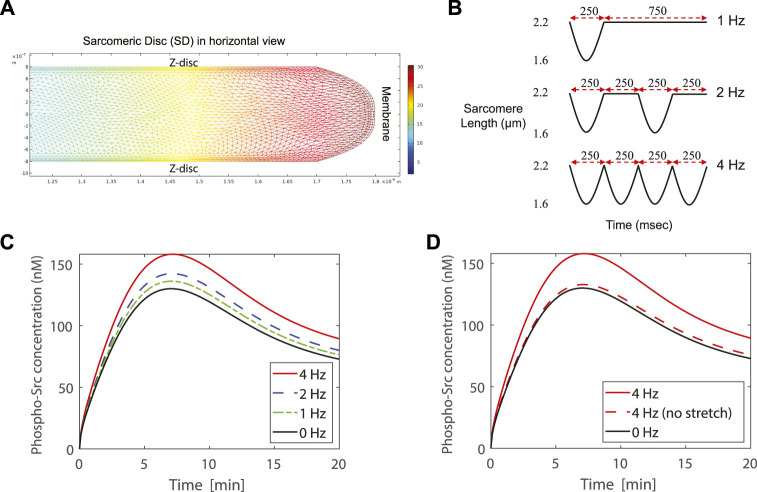
**(A)** The concentration of phospho-Src in the cytosol of one SD in the 4 Hz case at 25 s after PDGF stimulation. The SD is shown in horizontal view. **(B)** Contraction profiles of the modeled SD for 1, 2, and 4 Hz contraction frequency. **(C)** Simulation of a contracting SD, by modeling the lattice expansion together with membrane stretch, exhibits higher concentration of phospho-Src when SD beats with higher rates. Simulations show 21.5%, 9.4%, and 4.7% higher concentration of phospho-Src, respectively at 4, 2, and 1 Hz contraction frequencies, comparing to 0 Hz. **(D)** The dashed-red curve represents a case in which the lattice expansion completely compensates the cytosol displacement and there is no membrane stretch. In this case the peak value of the phospho-Src concentration is only 2.2% higher than the peak value of the 0 Hz case. Simulations demonstrate that membrane stretch has higher impact than lattice expansion in increasing the phospho-Src concentration. The lattice expansion with membrane stretch study are presented in dashed curves. The solid curves show the lattice expansion without membrane stretch.

In a simulation, first one cycle of motion is simulated using computational fluid dynamics with moving boundaries. As a result, the velocity field of the cytosol is obtained during this cycle. In a second step, this motion is repeatedly coupled with the transport of the substances both in the cytosol and in the membrane. The changes in concentration due to chemical reactions throughout the cell are simultaneously calculated. As an example, the concentration of phospho-Src in the cytosol of one SD in the 4 Hz case at 25 s after PDGF stimulation is shown in (Figure 5A). The further from the membrane, the lower the concentration.

The contraction-relaxation profiles of the modeled SD are represented in (Figure 5B) for 1, 2, and 4 Hz contraction frequencies. The case of 0 Hz is also drawn as a reference curve. The FRET microscopy (Figure 4A), in combination with our hypothesis, allowed us to focus on the Src activity at costameres, specifically. As demonstrated in (Figure 4A), Src kinase is mainly localized at the costameres in CMs. Therefore, in the modeled SD, we probed the evolution of phospho-Src concentration underneath the membrane at the costamere site (Figure 5C). All four curves show almost similar trajectories to our experimental results (Figure 4B). Simulations exhibit higher concentration of phospho-Src when sarcomere beats with higher rates. The maximum concentration of phospho-Src at 4, 2, and 1 Hz contraction frequencies, was 21.5%, 9.4%, and 4.7% higher, respectively, comparing to 0 Hz.

To distinguish the impact of membrane stretch from the impact of lattice expansion, we compared the reported SD model with an unrealistic model in which we assumed that the membrane is not elastic and cannot be stretched. This causes the Z-line lattice to be expanded more, to sustain the cytosolic volume constant. At the 4 Hz contraction of this assumed case, the peak value of the phospho-Src concentration was just 2.2% higher than the peak value of the 0 Hz case (Figure 5D). Thus, the membrane stretch has higher impact than lattice expansion in increasing the phospho-Src concentration.

## Discussion

The idea of cell shape having effects on cell physiology and pathology is probably not new and dates back to at least 1880s and 1890s, when Pflüger, Driesch and Hertwig reported about the manipulation of amphibian and invertebrate embryos and found that the regulation of mitotic orientations play a role in embryonic patterning (Pflüger, 1884; Driesch, 1891; Hertwig, 1893). This work eventually culminated in Hertwig’s Rule: the mitotic spindle bisects the cell perpendicular to its longest axis [for a recent review: (Cadart et al., 2014)].

In contrast, frequency dependent signaling has been proposed and detected in neurons [for a review (Ito and Schuman, 2008)]. While frequency dependent changes in calcium transients in cardiomyocytes are well known (Sipido et al., 1998), with calcium being an important intracellular mediator of contraction, frequency dependent effects on cell signaling did not receive much attention. Inspired by the work of Rangamani et al. and Kuo et al., we developed the concept of frequency dependent signaling in cardiomyocytes [please see for review: (Haftbaradaran Esfahani and Knoll, 2020b)].

Here we elaborate and provide mathematical evidence for the existence of a novel concept in physiology, namely that membrane shape translates into biochemical signaling, as suggested by a previous report (Rangamani et al., 2013), where cell signaling has been put in the context of membrane shape. Convex membranes foster ligand/receptor interaction and hence are expected to increase signaling, as long as the ligand remains extracellular. Elongated, elliptical cells are expected to increase their signaling activity *via* decreasing local membrane to volume ratios.

Our data whereby an increase in frequency increases the activation of Src kinase and where we show that Src kinase also plays an essential role in cardiomyocyte signaling provides evidence for the existence of membrane curvature related molecular mechanisms and that this mechanism displays relevance in biology and medicine.

The myocardium, where contracting myocytes periodically elongate and minimize non-cardiac cells such as cardiac fibroblasts and hence increase their sensitivity to receptor mediated effects, can lead to hypertrophy of these cells.

The situation in cardiac myocytes probably is more complex: cycles of contraction and relaxation will expose and de-expose costameres and the connecting membranes to the surrounding extracellular fluid and hence activate and de-activate this type of signaling (for example integrin mediated signaling such as Src kinase, focal adhesion kinase and integrin linked kinase) (Knoll et al., 2007). The membrane parts between costameres probably are bulging outwards which will increase membrane to cytosol ratios and hence increase signaling.

The frequency of beating hearts is expected to initiate very specific types of hypertrophic or atrophic signaling and as such affect cardiac metabolism, depending on whether costamere or membrane related signaling overweighs (Dixon and Spinale, 2009). This will also give rise to the differentiation of different types of cardiac hypertrophy initiated *via* different signal transduction cascades. For example, very low frequencies likely foster the development of different types of heart failure than very high frequencies (Alboni et al., 2001).

The development of heart failure in other models, such as the ones whereby an increase in cardiac preload or volume stretches cardiac myocytes and non-cardiac myocytes, and which cause eccentric hypertrophy, probably can be better explained by using the concept of local membrane in-homogeneities.

Every hypertrophy will lead to a decrease of surface to volume ratios but membrane ligand/receptor mediated signaling activity can be modified *via* frequency and hence cell shape. In the heart this is expected to lead to modifications of costamere related hypertrophic or atrophic stimuli. Thus hypertrophy can be regarded as part of a negative feedback loop aimed to equilibrate various membrane-mediated signaling pathways. The effects of local membrane in-homogeneities offer a novel molecular mechanism to explain various types of cardiac hypertrophy, which so far largely has been seen as a response to normalize wall stress.

## Limitations

Although the concept of locality of surface to volume ratios is attractive, it is still very general and to a large extent based on theoretical considerations which depend on specific reaction kinetics and diffusion probabilities, and which may differ from receptor to receptor, ligand to ligand and cell-type to cell-type. In addition, we primarily focused on Src kinase signaling, but did not take into consideration hundreds of other signal transduction cascades affecting cell survival or performance. Moreover, calcium is well known to be linked to the frequency of the beating cardiomyocyte and hence should be analyzed in the context of signaling as well.

Moreover, for reasons of simplicity, we assumed membrane changes underlie sinusoidal functions but in reality, this type of movement is very unlikely to occur in CMs.

Additional experimental proof is needed, particularly in the cardiovascular system. Nevertheless, this effect could be important for the heart.

## Summary

Mechanosensation, mechanotransduction and mechano-conversion are fundamental processes and are present in every living cell. These effects are particularly significant in dynamic cells such as cardiomyocytes and dynamic organs such as hearts. The addition of membrane curvature—and hence frequency-dependent, dynamic processes which differentially affect signaling and hence connect various types of information processing to cellular phenotypes is completely new and provides novel opportunities for basic science, translational medicine, drug development and therapy.

## Data Availability

The raw data supporting the conclusion of this article will be made available by the authors, without undue reservation.

## Appendix 1 List of reactions

### PDGF binds PDGFR

**Table:**

Name	Description	Expression	Units
J	Reaction rate	$K_{on}.c_{PDGF}.c_{PDGFR}-K_{off}.c_{PDGF-PDGFR}$	$molecules.\mu m^{-2}\cdot s^{-1}$
$K_{on}$	Forward rate constant	1	$\mu M^{-1}\cdot s^{-1}$
$K_{off}$	Reverse rate constant	0.01	$s^{-1}$
$c_{PDGF-PDGFR}$	PDGF-PDGFR complex concentration	Variable	$molecules\cdot \mu m^{-2}$

### Src association with PDGF-PDGFR complex

**Table:**

Name	Description	Expression	Units
J	Reaction rate	$K_{f}.c_{PDGF-PDGFR}.c_{Src}-K_{r}.c_{PDGF-PDGFR-Src}$	$molecules\cdot \mu m^{-2}\cdot s^{-1}$
$K_{f}$	Forward rate constant	1	$\mu M^{-1}\cdot s^{-1}$
$K_{r}$	Reverse rate constant	0.1	$s^{-1}$
$c_{PDGF-PDGFR-Src}$	PDGF-PDGFR-Src complex concentration	variable	$molecules\cdot \mu m^{-2}$

### Src activation (phosphorylation)

**Table:**

Name	Description	Expression	Units
J	Reaction rate	$\frac{V_{max\;}\;.c_{PDGF-PDGFR-Src}}{K_{m}+c_{PDGF-PDGFR-Src}}$	$\text{molecules}\cdot \mu m^{-2}\cdot s^{-1}$
$V_{max}$	Maximum reaction rate	$K_{cat}\cdot PTP$	$\text{molecules}\cdot \mu m^{-2}\cdot s^{-1}$
$K_{cat}$	Reaction constant	9	$\text{molecules}\cdot \mu m^{-2}\cdot \mu M^{-1}\cdot s^{-1}$
PTP	Species concentration	0.1	μM
$K_{m}$	Km (1/2 max)	0.1	$\text{molecules}\cdot \mu m^{-2}$
$c_{PDGF-PDGFR-Src}$	PDGF-PDGFR-Src complex concentration	Variable	$\text{molecules}\cdot \mu m^{-2}$

### Src dephosphorylation in cytosol

**Table:**

Name	Description	Expression	Units
J	Reaction rate	$\frac{V_{max}\;.c_{Phospho-Src}}{K_{m}+c_{Phospho-Src}}$	$\mu M\cdot s^{-1}$
$V_{max}$	Maximum reaction rate	0.3 ⋅Csk−active	$\mu M\cdot s^{-1}$
$K_{m}$	Km (1/2 max)	1.5	μM
$c_{Phospho-Src}$	Phospho-Src concentration	Variable	μM
